# Frequency and Duration of, and Risk Factors for, Diagnostic Delays Associated with Histoplasmosis

**DOI:** 10.3390/jof8050438

**Published:** 2022-04-23

**Authors:** Aaron C. Miller, Alan T. Arakkal, Scott H. Koeneman, Joseph E. Cavanaugh, George R. Thompson, John W. Baddley, Philip M. Polgreen

**Affiliations:** 1 Department of Internal Medicine, University of Iowa, Iowa City, IA 52242, USA; aaron-miller@uiowa.edu; 2 Department of Biostatistics, University of Iowa, Iowa City, IA 52242, USA; alan-arakkal@uiowa.edu (A.T.A.); scott-koeneman@uiowa.edu (S.H.K.); joe-cavanaugh@uiowa.edu (J.E.C.); 3 Department of Medicine, University of California, Davis, CA 95616, USA; grthompson@ucdavis.edu; 4 Department of Medicine, University of Maryland, Baltimore, MD 21201, USA; jbaddley@ihv.umaryland.edu; 5 Departments of Internal Medicine and Epidemiology, University of Iowa, Iowa City, IA 52242, USA

**Keywords:** histoplasmosis, diagnostic delays, diagnostic errors

## Abstract

Histoplasmosis is often confused with other diseases leading to diagnostic delays. We estimated the incidence, length of, and risk factors for, diagnostic delays associated with histoplasmosis. Using data from IBM Marketscan, 2001–2017, we found all patients with a histoplasmosis diagnosis. We calculated the number of visits that occurred prior to the histoplasmosis diagnosis and the number of visits with symptomatically similar diagnoses (SSDs). Next, we estimated the number of visits that represented a delay using a simulation-based approach. We also computed the number of potential opportunities for diagnosis that were missed for each patient and the length of time between the first opportunity and the diagnosis. Finally, we identified risk factors for diagnostic delays using a logistic regression model. The number of SSD-related visits increased significantly in the 97 days prior to the histoplasmosis diagnosis. During this period, 97.4% of patients had a visit, and 90.1% had at least one SSD visit. We estimate that 82.9% of patients with histoplasmosis experienced at least one missed diagnostic opportunity. The average delay was 39.5 days with an average of 4.0 missed opportunities. Risk factors for diagnostic delays included prior antibiotic use, history of other pulmonary diseases, and emergency department and outpatient visits, especially during weekends. New diagnostic approaches for histoplasmosis are needed.

## 1. Introduction

Histoplasmosis is an infectious disease caused by fungi of the genus *Histoplasma,* and the most common species is *Histoplasma capsulatum.* In the United States (US), *Histoplasma capsulatum* is endemic in the Ohio and Mississippi River valleys [1,2]. However, autochthonous cases have been reported outside these regions (e.g., Montana and California) [3,4], and a substantial proportion of cases occur beyond traditional regions of endemicity [5]. Because histoplasmosis is generally not a reportable disease [5,6], determining the true burden of histoplasmosis is difficult. However, between 500,000 and 3 million cases are estimated to occur annually in the US alone [7]. Histoplasmosis is the most common endemic fungal infection in the US [8], and it also occurs outside the US [5].

Most exposures to *Histoplasma capsulatum* do not result in illness [9,10]. Among people who do become symptomatic after exposure, the most common manifestation is a “influenza-like” illness that typically resolves in a matter of weeks without treatment [9,11]. However, the symptoms and the severity of histoplasmosis vary widely depending upon degree of inoculation and the host’s immune status [9,12,13]. Cases of histoplasmosis localized to the pulmonary system can cause an acute, subacute, or chronic illness [9]. The disease can also disseminate to the central nervous, gastrointestinal and hemopoietic systems. Histoplasmosis in its most severe form is progressive and disseminated and occurs predominantly among the immunocompromised, including people with AIDS, transplants or those receiving immunosuppressive medications (e.g., biologics and medications to prevent transplant rejection) [14,15,16].

Cases of histoplasmosis present with many different signs and symptoms. Manifestations most often include fevers, chills, weight loss, shortness of breath, lymphadenopathy, a dry cough and weight loss [9,11]. Furthermore, dissemination of the yeast can lead to central nervous, rheumatologic and gastrointestinal symptoms, as well as ulceration of mucus-membrane surfaces [17,18,19]. Involvement of the bone marrow can also lead to hematologic lab abnormalities. Because of the varied non-specific symptoms, the diagnosis of histoplasmosis is difficult. It can be confused with other infectious diseases (e.g., community-acquired pneumonia [20] and tuberculosis [21]), as well as non-infectious diseases (e.g., malignancies [22] and autoimmune disease [23,24]).

Difficulty diagnosing histoplasmosis can lead to delays in treatment. Failure to treat moderate-to-severe cases of histoplasmosis contributes to adverse outcomes ranging from prolonged symptoms to hospitalization and death [25]. In this paper, we estimate the incidence of diagnostic delays associated with histoplasmosis, estimate the average length of delays associated with histoplasmosis, and identify specific risk factors for diagnostic delays. In all cases, we specifically focus on cases of histoplasmosis requiring an antifungal treatment.

## 2. Materials and Methods

### 2.1. Data Sources and Study Population

Longitudinal insurance claims from the IBM Marketscan Research Databases from 2001 to 2017 were used in this study, including both commercial claims and Medicare supplemental claims. Within these databases, over 195 million people are represented. Data include commercial insurance claims for outpatient prescriptions and visits to outpatient, emergency department and inpatient facilities.

We identified all patients with a histoplasmosis diagnosis using diagnosis codes 115.XX (ICD-9-CM) and B39.X (ICD-10-CM). The index diagnosis date was defined as the first histoplasmosis diagnosis for a given patient. To account for the potentially long disease course of histoplasmosis, we included patients with one year of continuous enrollment prior to their index diagnosis. Furthermore, we excluded patients that had a histoplasmosis retinitis diagnosis in the year prior to their index histoplasmosis diagnosis (ICD-9-CM: 115.02, 115.12, and 115.92; ICD-10-CM: B39.4, B39.5, B39.9, and H32). Additionally, we applied two inclusion criteria to further restrict our study population: (1) patients who were treated for histoplasmosis within 90 days of the index diagnosis (i.e., treatment with fluconazole, itraconazole, voriconazole, posaconazole, or amphotericin); or (2) patients who had an inpatient index histoplasmosis diagnosis and died within 7 days of their diagnosis.

### 2.2. Statistical Analysis

Our analysis aimed to address two primary objectives: (1) determine the frequency and duration of diagnostic delays associated with histoplasmosis, and (2) estimate risk factors for a potential missed diagnostic opportunity. We used a previously developed simulation-based approach to identify missed diagnostic opportunities that employs a case-crossover type design, where each case patient serves as their own control during the period before presence of clinical disease (i.e., histoplasmosis) occurs [26,27]. This approach builds upon other studies to identify delays in longitudinal administrative claims data [28,29]. Specifically, this method estimates the number of excess visits that are associated with signs and symptoms of the disease and occur before the index diagnosis. We define excess visits to be healthcare visits that exceed the quantity of care expected to occur if histoplasmosis were not present. A bootstrapping procedure is then used to compute metrics associated with diagnostic delays. Additional details of this approach can be found in [26,27].

Specifically, we start by identifying *symptomatically similar diagnoses* (SSDs) occurring before the index histoplasmosis diagnosis, which we define as diagnoses with signs and symptoms similar to histoplasmosis (e.g., cough, fever, and fatigue). Table S1 presents the SSDs and the corresponding ICD-9/10-CM codes used for this analysis, and Table S2 provides descriptions. After identifying all SSD visits in the year before the histoplasmosis diagnosis, we apply the CUMSUM change-point-finding approach to identify the time period prior to diagnosis when signs and symptoms of histoplasmosis are significantly elevated. We define this period of elevated SSD visits as the diagnostic opportunity window, where missed diagnostic opportunities may occur, and use the time prior to this window as the control period in our crossover analysis where the expected pattern of care (i.e., in absence of disease) can be estimated.

**Figure 1:**
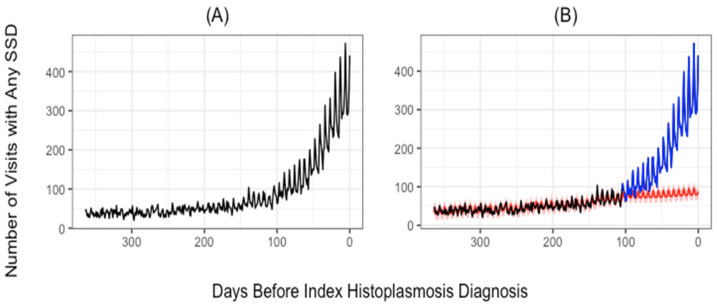
Trends in observed and expected number of symptomatically similar diagnoses (SSD)-related visits. The number of visits with any SSD-related diagnosis (vertical axis) is plotted for the number of days prior to the index histoplasmosis diagnosis (horizontal axis). Observed SSD-related visits are given in panel (**A**). In panel (**B**), the red line was estimated using the data collected before the change point and gives the trend in expected visits. The blue line represents the actual number of visits after the change point. Possible diagnostic opportunities are represented by the area between the blue line and the red line.

The expected pattern of SSD visits was estimated by fitting a time-series model which incorporated first-order autoregressive and moving average components, additive effects for day of the week, as well as a linear trend. This was done to account for serial correlation as well as the 7 day periodic pattern present in the data. We then compute the number of missed opportunities as the excess number of visits during the diagnostic opportunity window, where excess visits are the number of observed visits minus the number of expected visits. In an attempt to be conservative, we defined the expected trend as the upper end of a 95% prediction interval. Figure 1 depicts this change point, crossover periods and estimated trends. Finally, to evaluate the incidence of missed diagnostic opportunities, the number of patients who experienced missed opportunities, and the duration of diagnostic delays, we employ a bootstrap-based procedure to repeatedly select which patient visits represent missed opportunities and compute the frequency and length of diagnostic delays in these patients. We repeated this procedure 25,000 times to estimate 95% confidence intervals for all estimates of interest.

Estimating Potential Diagnostic-Delay Risk Factors: Using a logistic regression model, we conducted an exploratory analysis to evaluate possible risk factors for a missed diagnostic opportunity. Specifically, any SSD-related visit in the diagnostic opportunity window was assumed to be a potential missed diagnostic opportunity and was designated “1”. Alternatively, the index histoplasmosis visit was designated “0”. We evaluated patient-, context-, and setting-related risk factors for diagnostic delays. Patient-related factors included sex, age, rurality, and concurrent HIV diagnosis. The following clinical factors were evaluated as markers of pre-existing pulmonary conditions before the diagnostic opportunity window: (1) underlying asthma, COPD, or interstitial pulmonary diseases (IPD); (2) chest X-ray or a chest CT; and (3) receipt of respiratory antibiotics. (The list of respiratory antibiotics are given in Table S3). Context-specific factors considered include the healthcare setting of a visit (i.e., inpatient, outpatient, or emergency department (ED)). (ICD and CPT codes for these factors are given in Table S4). Because multiple visits that occur on the same day often represent one episode of care, visits were aggregated by day. However, indicators for all combinations of daily visits (e.g., inpatient only, ED and inpatient) were included. We also considered metropolitan statistical area (MSA), state and regional incidence of histoplasmosis, as clinician awareness and disease prevalence are often inversely associated with diagnostic delays [26]. We considered multiple ways to measure local histoplasmosis incidence along with different model specifications (see Table S5). We also included the month and year of the index diagnosis and SSD visit. Standard errors were used to compute Wald-type 95% confidence intervals for the logistic regression analysis.

Sensitivity Analyses: Insurance claims based on diagnostic codes do not contain all signs and symptoms that are present during clinic visits. Thus, if symptoms are not recorded, we most likely underestimate the number of visits that represent missed opportunities. Therefore, we repeated all of our analyses considering all visits as potential diagnostic opportunities, whether there was an SSD present or not.

## 3. Results

**Figure 2:**
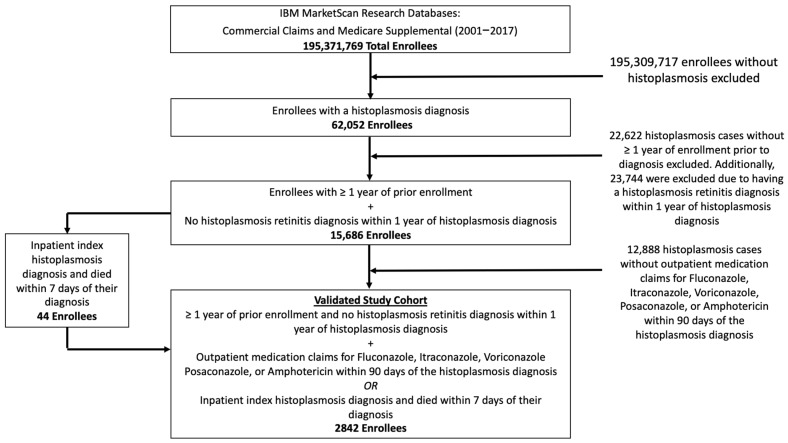
Inclusion and exclusion criteria and counts of included and excluded patients.

Figure 2 presents the study cohort flow diagram. Between 2001 and 2017, we identified a total of 62,052 individuals with a histoplasmosis diagnosis. Applying all eligibility criteria (i.e., continuous enrollment for one year prior to diagnosis, medication treatment, death within 7 days of index, and excluding Histoplasmosis retinitis), resulted in a final study cohort of 2842 enrollees. Table 1 presents the baseline characteristics of the study cohort.

Figure 1A presents the number of SSD visits occurring in the year before the index histoplasmosis diagnosis. Figure S1 depicts similar patterns for both all visits and SSD visits by healthcare-setting type. Figure S2 presents trends for five categories of SSD diagnoses utilized in the analysis. A consistent pattern is apparent across nearly all settings: SSD visits gradually increase from 1 year before the index diagnosis to approximately 100 days before the index diagnosis, after which a dramatic spike in SSD visits is observed just prior to histoplasmosis diagnosis.

Of the 2842 enrollees identified, 2819 (99.2%) patients had at least one healthcare visit in the 365 days before the index histoplasmosis diagnosis. We observed 2727 (96.0%) patients with at least one SSD visit in the year before the index diagnosis. More than one-third (36.9%) of visits during the year before the index diagnosis involved at least one SSD. Table S6 presents the breakdown of the number of patients and number of visits by SSD category in the year before the index diagnosis and during the delay window. The most common SSD category in the year prior to the index was symptom-based diagnoses, occurring in 2385 (83.9%) patients, and the least common SSD category was alternative gastrointestinal-based diagnoses, occurring in 437 (15.4%) of patients. Numbers of patients and visits for individual diagnoses are in Table S2.

The CUSUM change-point analysis identified the period between 1 and 97 days prior to the index diagnosis to be the period with excess SSD visits (i.e., the diagnostic opportunity window). The trend lines for both observed and expected SSD visits were estimated using change-point analysis and are presented in Figure 1B. A total of 2562 (90.1%) patients had at least one SSD during the diagnostic opportunity window. We observed 18,886 SSD visits during the delay opportunity window, of which 11,298 (59.8%) were estimated to be missed opportunities, occurring in 9755 (86.3%) outpatient, 557 (4.9%) inpatient, and 986 (8.7%) ED settings.

Table 2 presents the results from the bootstrapping approach to simulate individual visits and to estimate the number of missed opportunities experienced by each patient. We estimated that before the index diagnosis, 2355 (82.9%) (CI: 2300–2411 (80.9–84.8%)) patients experienced at least one missed diagnostic opportunity. Of the patients who experienced at least one missed opportunity, on average, they experienced 4.03 (CI: 3.73–4.33) visits representing missed opportunities, with the majority, 3.48 (CI: 3.21–3.74), occurring in the outpatient setting. Furthermore, the mean duration of delays, among those delayed, was estimated to be 39.46 days (CI: 36.04–44.00). Approximately one-third (35.2% (CI: 30.4–39.7%)) of patients who were delayed experienced a delay that lasted 50 days or more.

We conducted sensitivity analyses and included all visits with or without an SSD. When utilizing all visits instead of just SSD visits, the estimated diagnostic opportunity window started 105 days before diagnosis. A total of 2773 patients (97.6%) had a visit during the delay window (i.e., 1 to 105 days before the index diagnosis) for any reason. A total of 39,230 visits were observed to occur during the delay window. Of the 39,230 visits, 14,434 (36.8%) were estimated to be missed opportunities. Additionally, we estimated that 2647 (93.1%) (CI: 2613–2679 (91.9–94.3%)) patients had one or more missed diagnostic opportunities before the index diagnosis. Furthermore, on average, patients experienced 5.45 (CI: 5.05–5.87) missed diagnostic opportunities. Additionally, the average delay lasted 54.42 days (CI: 49.22–60.28).

The likelihood of experiencing a potential missed opportunity is shown in Table 3. We found that many patient-associated factors were associated with an increased likelihood of a potential missed diagnostic opportunity. Patients with a history of COPD (OR: 1.298 (CI: 1.130–1.491)) were more likely to experience a missed opportunity. Similarly, patients who had received chest imaging in the year prior to diagnosis but before the diagnostic opportunity window were more likely to experience a missed opportunity (OR: 1.602 (CI: 1.457–1.761)) for chest CT and 2.363 (CI: 2.126–2.625) for chest X-ray). Furthermore, patients who received respiratory antibiotics during the diagnostic opportunity window were significantly more likely to experience a missed opportunity (OR: 1.285 (CI: 1.173–1.408)).

Healthcare-setting- and context-specific factors were also significantly associated with missed opportunities. Missed opportunities occurred often during weekend visits (OR: 1.855 (CI: 1.514–2.273)). Missed opportunities were less likely to occur on days involving inpatient visits compared to outpatient settings alone. In contrast, visits to the ED appeared to increase the risk of a missed opportunity. Missed opportunities were more common when patients only visited the ED (OR: 6.784 (CI: 3.892–11.823)) and less common when patients only visited inpatient settings (OR: 0.129 (CI: 0.111–0.151)).

Finally, we did not find a consistent significant relationship between the incidence of histoplasmosis in a patient’s location and their risk for experiencing a missed opportunity. Table S5 provides the results of the regression analyses where we explored various ways to measure local histoplasmosis incidence. We considered histoplasmosis incidence measured at an MSA-level, state-level and a regional level, using incidence measured via our study population as well as data from prior publications or CDC reporting regions. Across the various models, regional histoplasmosis incidence was not associated with missed opportunities. Therefore, we omitted histoplasmosis incidence from our final model. Table S7 also presents results of the risk factor model stratified by which ICD version the index diagnosis was coded as (i.e., 9 vs. 10); in general, we do not find major differences by ICD version.

## 4. Discussion

In this study, we investigated the incidence and length of diagnostic delays associated with histoplasmosis. Because finding a suitable control group is difficult (e.g., those hospitalized for another reason), we used a case-crossover design, where each histoplasmosis case served as their own control. Within our cohort, we identified a significant increase in the number of visits associated with SSDs in the 97 days prior to the histoplasmosis diagnosis, indicating that visits associated with histoplasmosis start increasing over three months prior to diagnosis. During these 97 days (i.e., our estimated diagnostic opportunity window), 97.4% of patients had any healthcare visit, and 90.1% had at least one visit that we considered an SSD. Because some of these visits could be coincidental, we used a simulation-based approach to estimate missed diagnostic opportunities and found that 82.9% of patients with histoplasmosis experienced one missed opportunity or more. Patients experiencing a missed opportunity had an average delay lasting 39.5 days and an average of 4.0 missed opportunities from their first presentation; over one-third of these patients were delayed for ≥50 days.

Diagnostic delays associated with histoplasmosis are commonly reported in the literature, and the disease can present with many different symptoms that are common to a wide range of other diseases [17,18,23]. For example, histoplasmosis can mimic the presentation of other respiratory infections [20], malignancies [22], and autoimmune diseases [9,24]. While delays are commonly reported risk factors for these diagnostic delays are relatively unknown. Our results highlight multiple risk factors that could help inform future interventions to decrease diagnostic delays of histoplasmosis. In addition to risk factors, most previous research has not focused on estimating the number and length of missed opportunities.

We identified multiple potential risk factors for missed opportunities to diagnose histoplasmosis. For example, we found that missed opportunities were more strongly associated with healthcare visits that occurred on weekends. Many different adverse healthcare outcomes are linked to weekend-based healthcare [30,31,32], thus it is not surprising that diagnostic delays are also more common. In terms of practice settings, missed opportunities were more commonly associated with outpatient visits and ED visits compared to hospitalizations. Most investigations for diagnostic delays do not consider different practice settings, but those that have, also found outpatient and ED settings more likely to be associated with delays compared to inpatient settings [33]. Unlike some other infectious diseases, there are diagnostic tests that aid in the diagnosis of histoplasmosis (e.g., urine and serum antigen tests) [34]. Testing availability may be more limited in outpatient and ED settings that are not attached to hospitals as well as during weekends. Point-of-care diagnostic tests are not routinely available for histoplasmosis, and a sensitive point-of-care test would decrease diagnostic delays and would be especially useful in immunosuppressed patients and in regions where histoplasmosis is endemic. Additionally, the aggressive use of diagnostic tests to rule out infections other than histoplasmosis (e.g., bacterial or viral infections) will help accelerate the diagnosis of histoplasmosis.

Beyond demographic- and practice-based risk factors, we also investigated the potential role of cognitive errors as a risk factor for delays. Cognitive errors include failing to consider a diagnosis or failure to give the appropriate weight to alternative diagnoses [35,36]. Because we cannot directly measure cognitive errors, we investigated how a patient’s medical history is related to the time it takes to make a diagnosis of histoplasmosis. We found that diagnostic delays were significantly more common in people with pre-existing pulmonary diseases (e.g., COPD, or remote history of chest imaging). This finding is consistent with a cognitive error commonly referred to as premature closure [37,38]. Specifically, physicians are less likely to make an earlier diagnosis of histoplasmosis in patients with pre-existing pulmonary disease because they are more likely to attribute a patient’s pulmonary symptoms to an exacerbation of a prior known pulmonary disease rather than an alternative diagnosis (e.g., histoplasmosis). Local incidence of disease has been found to be inversely associated with risk for delay for other infectious diseases such as tuberculosis [26]. However, we did not find strong evidence that the incidence of histoplasmosis in a patient’s location was associated with risk for diagnostic delays.

The inappropriate use of antibiotics has been associated with diagnostic delays for several different infections [39]. Specific examples include tuberculosis [33] and endocarditis [40], and patients may transiently improve, leading to a delayed presentation following the receipt of inappropriately prescribed antimicrobials. We found that the prescribing of respiratory antibiotics during our diagnostic opportunity window for histoplasmosis was associated with a diagnostic delay. Because antibiotics typically used to treat bacterial pathogens are not effective against histoplasmosis, this delay must be attributable to patients or their healthcare providers wanting to wait a sufficient period to allow for a possible therapeutic effect. The inappropriate use of antimicrobials is common [41,42,43], and our results provide another reason to use antimicrobials more judicially, especially in areas of endemicity and among immunosuppressed patients, who may be more likely to develop severe cases of histoplasmosis.

This study has multiple major limitations. First, we rely on observational administrative claims data and corresponding diagnosis codes to identify the index histoplasmosis events and symptomatic visits prior to a diagnosis. Histoplasmosis presentation is varied. Histoplasmosis can be acute, chronic, or disseminated, and administrative claims do not differentiate among all possible clinical syndromes associated with histoplasmosis. Accordingly, we did not attempt to compare delays between subacute, chronic or disseminated illness when estimating the duration and frequency of, and risk factors for, diagnostic delays. However, for our case definition, we not only required a diagnostic code for histoplasmosis but also a prescription for an antifungal. Using this approach eliminated cases that did not require treatment or were not active cases (e.g., an incidental finding on a lung biopsy). This eliminated a large portion of cases that were identified using only diagnostic codes.

Second, we do not have access to lab data to confirm histoplasmosis diagnoses nor do we have access to clinical notes to confirm the presence of related symptoms. It is possible that we are missing symptomatic visits where symptoms are not recorded as a diagnosis code. However, we conducted a sensitivity analysis by including all visits as potential missed opportunities; this resulted in a greater proportion of patients estimated to experience a missed opportunity, but the mean number of missed opportunities and duration of delays were not markedly different. Another limitation of our study is that our dataset represents a commercially insured population and that we do not have access to detailed demographic information (e.g., race and ethnicity). Thus, our estimates for the frequency and duration of missed opportunities may be underestimates, especially for individuals without access to health insurance, those with less generous insurance coverage or for individuals with less access to care. Additionally, because we focused on patients that were treated with antifungals, we are unable to estimate delays among the substantial number of cases that resolve without treatment. Finally, in some cases, some of the SSDs we identified, instead of symptoms of histoplasmosis, may have represented risk factors, and this could potentially affect some of our findings.

## 5. Conclusions

Despite the limitations associated with our results, we clearly demonstrate that people diagnosed with histoplasmosis frequently experience diagnostic delays. Furthermore, we identified multiple potential risk factors for diagnostic delays, including prior antibiotic use, history of other pulmonary diseases, and healthcare visits other than hospitalization and during weekends. Both the delays we documented and the potential risk factors we identified highlight the need for new diagnostic approaches, and some of this work is currently underway [44]. Our results highlight the importance of future efforts to improve diagnostic efficiency for this important endemic fungal infection.

## Figures and Tables

**Table 1:** Baseline characteristics of study cohort.

Variable	Total Patients (% of Patients)
Age at Diagnosis	
<18	147 (5.2%)
18–35	430 (15.1%)
36–45	490 (17.2%)
46–55	689 (24.2%)
56–65	751 (26.4%)
>65	335 (11.8%)
Sex	
Male	1418 (49.9%)
Female	1424 (50.1%)
Enrollment time prior to index (years)	
Mean	4.6
Median	3.5
Range	1.0–16.9
Count ≤ 1.5 years	348 (12.2%)
Count ≤ 2 years	668 (23.5%)
Count ≤ 3 years	1180 (41.5%)
Count > 3 years	1662 (58.5%)
Region	
Rural	729 (25.7%)
Urban	2096 (73.8%)
Missing	17 (0.6%)
Month of index diagnosis	
January	265 (9.3%)
February	266 (9.4%)
March	260 (9.1%)
April	241 (8.5%)
May	247 (8.7%)
June	231 (8.1%)
July	227 (8.0%)
August	219 (7.7%)
September	227 (8.0%)
October	214 (7.5%)
November	223 (7.8%)
December	222 (7.8%)

**Table 2:** Number of delays per patient (out of all patients).

Metric/Category	Count (Percentage of All Patients)/Mean	95% CI (from Bootstrapping)
Number of missed opportunities		
0	487 (17.1%)	431–542 (15.2–19.1%)
>=1	2355 (82.9%)	2300–2411 (80.9–84.8%)
>=2	1960 (69.0%)	1871–2045 (65.8–72.0%)
>=3	1550 (54.5%)	1435–1660 (50.5–58.4%)
>=4	1159 (40.8%)	1035–1282 (36.4–45.1%)
>=5	825 (29.0%)	705–946 (24.8–33.3%)
Mean—Overall	4.03	3.73–4.33
Mean—Outpatient	3.48	3.21–3.74
Mean—Inpatient	0.20	0.18–0.22
Mean—ED	0.35	0.31–0.39
Duration of delays (days)		
>=0	2355 (100.0%)	2300–2411 (NA)
>=10	2116 (88.9%)	2053–2166 (87.4–90.2%)
>=20	1802 (75.7%)	1718–1865 (73.3–77.7%)
>=30	1475 (61.9%)	1390–1555 (59.0–65.0%)
>=40	1174 (49.7%)	1065–1280 (45.6–53.4%)
>=50	833 (35.2%)	713–954 (30.4–39.7%)]
>=60	477 (20.0%)	463–492 (19.4–20.6%)
>=70	401 (17.0%)	279–517 (11.9–21.6%)
>=80	117 (5.0%)	61–175 (2.6–7.4%)
>=90	28 (1.2%)	17–53 (0.7–2.3%)
Mean	39.46	36.04–44.00

Note: ED = emergency department.

**Table 3:** Regression results for the likelihood of a potential missed opportunity.

Variable	Adjusted Odds Ratio	95% CI	*p*-Value
Weekend (visits that occurred on a Saturday or Sunday)	1.855	1.514, 2.273	<0.001
Female Sex	0.984	0.900, 1.075	0.716
Age			
<18	REF	REF	REF
18–35	1.048	0.834, 1.316	0.689
36–45	1.142	0.911, 1.430	0.249
46–55	1.169	0.941, 1.453	0.159
56–65	1.192	0.959, 1.480	0.113
>65	1.279	1.010, 1.621	0.041
Settings visited			
Outpatient only	REF	REF	REF
All three (inpatient, outpatient, and ED)	0.158	0.102, 0.246	<0.001
ED only	6.784	3.892, 11.823	<0.001
Inpatient only	0.129	0.111, 0.151	<0.001
Inpatient and ED	0.149	0.110, 0.202	<0.001
Inpatient and outpatient	0.134	0.114, 0.158	<0.001
Outpatient and ED	2.898	1.836, 4.573	<0.001
Urban vs. not urban	1.019	0.920, 1.129	0.715
Asthma prior to change point	1.161	0.983, 1.371	0.079
COPD prior to change point	1.298	1.130, 1.491	<0.001
ILD prior to change point	1.591	0.811, 3.122	0.177
HIV prior to index	0.845	0.670, 1.065	0.154
Chest CT prior to change point	1.602	1.457, 1.761	<0.001
Chest X-ray prior to change point	2.363	2.126, 2.625	<0.001
Respiratory antibiotics between change point and 1 day prior to index	1.285	1.173, 1.408	<0.001

Note: Model was also adjusted for year and month of SSD/index visit. ED = emergency department; COPD = chronic obstructive pulmonary disease; IPD = interstitial pulmonary disease; HIV = human immunodeficiency virus; CT = computerized tomography. Potential missed opportunities are defined as patients with an SSD-related healthcare visit during the diagnostic opportunity window; this may include visits that represent a true missed opportunity (i.e., histoplasmosis is present prior to index diagnosis) and coincidental visits.

## Data Availability

The IBM MarketScan Research Databases used for this study are widely used research databases that are available for purchase. These databases are proprietarily owned by IBM and can be purchased by contacting IBM Watson Health. We are not legally permitted to release these data to researchers outside of the University of Iowa; however, these data can be obtained from IBM Watson Health. The code used for these methods is available (https://github.com/aarmiller/delaySim, accessed on 20 April 2022) as is an accompanying methods paper (MedRxiv, https://www.medrxiv.org/content/10.1101/2021.10.22.21265386v1, accessed on 20 April 2022).

## Supplementary Materials

The following supporting information can be downloaded at: https://www.mdpi.com/article/10.3390/jof8050438/s1.
