# Supplementary material for: Frequency and Duration of, and Risk Factors for, Diagnostic Delays Associated with Histoplasmosis
Source: J Fungi (Basel). 2022 Apr 23;8(5):438. doi: 10.3390/jof8050438 (PMC9143509; doi:10.3390/jof8050438)
Supplement: Supplementary file 1 [file jof-08-00438-s001.zip › jof-1596816-supplementary.pdf]

Supplementary Material

Supplementary Table S1 - List of symptomatically similar diagnoses (SSDs) used to identify potential diagnostic opportunities

Category	ICD-9-CM	ICD-10-CM
Symptom-based diagnoses	780.6, 780.60, 780.79, 783.21, 780.8, 784.0, 786.05, 786.07, 786.2, 786.3, 786.30, 786.50, 786.52, 786.59, 787.02, 787.91, 789.00, 789.01, 789.06, 789.07	R04.2, R05, R06.00, R06.02, R06.09, R06.2, R06.3, R06.83, R06.89, R07.1, R07.81, R07.82, R07.89, R07.9, R10.11, R10.13, R10.84, R10.9, R11.0, R19.7, R50.9, R51, R53.1, R53.81, R53.83, R61, R63.4
Alternative infectious disease-based diagnoses	010, 011, 012, 013, 014, 015, 016, 017, 018, 079.99, 461.9, 462, 465.8, 465.9, 466.0, 473.9, 482.9, 486, 490, 491.9, 513.0, 599.0, 790.8	A15, A17, A18, A19, B34.9, B97.89, G93.3, J01.90, J02.9, J06.9, J15.9, J18.2, J18.9, J20.9, J32.9, J40, J85.1, J85.2, N39.0, R50.2
Alternative cardiopulmonary-based diagnoses	135, 162.3, 162.5, 162.9, 212.3, 235.7, 239.1, 423.9, 428.0, 491.20, 491.21, 492.0, 492.8, 493.20, 493.22, 493.90, 496, 511.0, 511.1, 511.89, 514, 515, 516.9, 518.51, 518.53, 518.81, 518.84, 518.89, 786.09	C34.10, C34.11, C34.30, C34.90, D14.30, D38.1, D49.1, D64.9, D86.0, D86.9, I31.9, I50.9, I88.9, J42, J43.9, J44.1, J44.9, J45.909, J45.998, J81.1, J82, J84.10, J84.89, J84.9, J85.0, J86.9, J91.8, J93.83, J94.1, J94.8, J94.9, J96.00, J96.01, J96.21, J96.90, J98.19, J98.4, R04.9, R09.1
Alternative gastrointestinal-based diagnosis	530.81, 560.9	K21.9, K50.90, K56.60, K80.20
Testing Imaging or Physical Examination-based diagnoses	276.1, 284.19, 285.9, 287.5, 288.60, 289.3, 429.3, 511.9, 512.8, 512.89, 518.0, 518.3, 555.9, 574.20, 729.81, 782.2, 783.21, 784.2, 785.6, 786.6, 789.2, 790.4, 793.1, 793.11, 793.19, 799.02, V74.1	D61.818, D69.6, D72.829, E87.1, I51.7, J90, J93.9, J98.11, R09.02, R16.1, R22.0, R22.1, R22.2, R59.0, R59.1, R59.9, R63.4, R74.0, R91.1, R91.8, Z11.1

Supplementary Table S2 - Count of specific symptomatically similar diagnoses (SSDs)

ICD Code	ICD Version	ICD Description	Entire Study Period (1-365 Days Prior to histoplasmosis diagnosis)		Delay Opportunity Window (1-97 Days Prior to histoplasmosis diagnosis)	
			Total Visits with SSD (% of all visits in window)	Total Patients with SSD (% of all patients)	Total Visits with SSD (% of all visits in window)	Total Patients with SSD (% of all patients)
Alternative Cardio-pulmonary Based Diagnoses						
518.89	9	Other diseases of lung, not elsewhere classified	3452 (7.7%)	1281 (46.1%)	2567 (9.2%)	1139 (41.0%)
496	9	Chronic airway obstruction, not elsewhere classified	1395 (3.1%)	300 (10.8%)	604 (2.2%)	230 (8.3%)
786.09	9	Other respiratory abnormalities	856 (1.9%)	512 (18.4%)	501 (1.8%)	354 (12.7%)
493.9	9	Asthma, unspecified type, unspecified	632 (1.4%)	266 (9.6%)	302 (1.1%)	170 (6.1%)
515	9	Postinflammatory pulmonary fibrosis	623 (1.4%)	401 (14.4%)	452 (1.6%)	329 (11.8%)
162.9	9	Malignant neoplasm of bronchus and lung, unspecified	591 (1.3%)	184 (6.6%)	380 (1.4%)	158 (5.7%)
428	9	Congestive heart failure, unspecified	379 (0.8%)	114 (4.1%)	148 (0.5%)	69 (2.5%)
491.21	9	Obstructive chronic bronchitis with (acute) exacerbation	314 (0.7%)	101 (3.6%)	141 (0.5%)	64 (2.3%)

135	9	Sarcoidosis	289 (0.6%)	90 (3.2%)	182 (0.7%)	77 (2.8%)
492.8	9	Other emphysema	264 (0.6%)	110 (4.0%)	143 (0.5%)	82 (3.0%)
518.81	9	Acute respiratory failure	233 (0.5%)	102 (3.7%)	141 (0.5%)	76 (2.7%)
518.84	9	Acute and chronic respiratory failure	184 (0.4%)	20 (0.7%)	42 (0.2%)	12 (0.4%)
162.3	9	Malignant neoplasm of upper lobe, bronchus or lung	179 (0.4%)	47 (1.7%)	98 (0.4%)	41 (1.5%)
235.7	9	Neoplasm of uncertain behavior of trachea, bronchus, and lung	142 (0.3%)	97 (3.5%)	127 (0.5%)	90 (3.2%)
423.9	9	Unspecified disease of pericardium	125 (0.3%)	58 (2.1%)	96 (0.3%)	48 (1.7%)
511	9	Pleurisy without mention of effusion or current tuberculosis	120 (0.3%)	94 (3.4%)	78 (0.3%)	60 (2.2%)
212.3	9	Benign neoplasm of bronchus and lung	116 (0.3%)	104 (3.7%)	93 (0.3%)	86 (3.1%)
239.1	9	Neoplasm of unspecified nature of respiratory system	112 (0.3%)	86 (3.1%)	95 (0.3%)	73 (2.6%)
162.5	9	Malignant neoplasm of lower lobe, bronchus or lung	73 (0.2%)	31 (1.1%)	55 (0.2%)	28 (1.0%)
491.2	9	Obstructive chronic bronchitis without exacerbation	68 (0.2%)	42 (1.5%)	31 (0.1%)	25 (0.9%)
493.2	9	Chronic obstructive asthma, unspecified	62 (0.1%)	38 (1.4%)	24 (0.1%)	21 (0.8%)
514	9	Pulmonary congestion and hypostasis	55 (0.1%)	48 (1.7%)	37 (0.1%)	36 (1.3%)

493.22	9	Chronic obstructive asthma with (acute) exacerbation	37 (0.1%)	16 (0.6%)	13 (0.0%)	7 (0.3%)
492	9	Emphysematous bleb	22 (0.0%)	18 (0.6%)	11 (0.0%)	9 (0.3%)
511.89	9	Other specified forms of effusion, except tuberculous	11 (0.0%)	8 (0.3%)	7 (0.0%)	7 (0.3%)
516.9	9	Unspecified alveolar and parietoalveolar pneumonopathy	8 (0.0%)	8 (0.3%)	5 (0.0%)	5 (0.2%)
518.51	9	Acute respiratory failure following trauma and surgery	7 (0.0%)	7 (0.3%)	3 (0.0%)	3 (0.1%)
511.1	9	Pleurisy with effusion, with mention of a bacterial cause other than tuberculosis	5 (0.0%)	5 (0.2%)	4 (0.0%)	4 (0.1%)
J44.9	10	Chronic obstructive pulmonary disease, unspecified	129 (0.3%)	25 (0.9%)	53 (0.2%)	20 (0.7%)
J98.4	10	Other disorders of lung	98 (0.2%)	50 (1.8%)	77 (0.3%)	42 (1.5%)
J84.10	10	Pulmonary fibrosis, unspecified	44 (0.1%)	23 (0.8%)	27 (0.1%)	17 (0.6%)
C34.90	10	Malignant neoplasm of unspecified part of unspecified bronchus or lung	38 (0.1%)	9 (0.3%)	19 (0.1%)	8 (0.3%)
J84.9	10	Interstitial pulmonary disease, unspecified	37 (0.1%)	8 (0.3%)	27 (0.1%)	7 (0.3%)
D64.9	10	Anemia, unspecified	35 (0.1%)	18 (0.6%)	23 (0.1%)	14 (0.5%)
J45.909	10	Unspecified asthma, uncomplicated	35 (0.1%)	16 (0.6%)	18 (0.1%)	11 (0.4%)

J44.1	10	Chronic obstructive pulmonary disease with (acute) exacerbation	32 (0.1%)	8 (0.3%)	10 (0.0%)	5 (0.2%)
D86.9	10	Sarcoidosis, unspecified	23 (0.1%)	6 (0.2%)	13 (0.0%)	5 (0.2%)
J96.01	10	Acute respiratory failure with hypoxia	22 (0.0%)	10 (0.4%)	20 (0.1%)	8 (0.3%)
J96.21	10	Acute and chronic respiratory failure with hypoxia	19 (0.0%)	6 (0.2%)	13 (0.0%)	4 (0.1%)
I50.9	10	Heart failure, unspecified	17 (0.0%)	4 (0.1%)	8 (0.0%)	3 (0.1%)
J43.9	10	Emphysema, unspecified	16 (0.0%)	10 (0.4%)	9 (0.0%)	6 (0.2%)
C34.11	10	Malignant neoplasm of upper lobe, right bronchus or lung	12 (0.0%)	4 (0.1%)	12 (0.0%)	4 (0.1%)
D86.0	10	Sarcoidosis of lung	8 (0.0%)	7 (0.3%)	5 (0.0%)	4 (0.1%)
J85.0	10	Gangrene and necrosis of lung	7 (0.0%)	6 (0.2%)	7 (0.0%)	6 (0.2%)
R09.1	10	Pleurisy	7 (0.0%)	5 (0.2%)	6 (0.0%)	5 (0.2%)
J91.8	10	Pleural effusion in other conditions classified elsewhere	6 (0.0%)	4 (0.1%)	5 (0.0%)	3 (0.1%)
J96.00	10	Acute respiratory failure, unspecified whether with hypoxia or hypercapnia	6 (0.0%)	6 (0.2%)	6 (0.0%)	6 (0.2%)
J84.89	10	Other specified interstitial pulmonary diseases	5 (0.0%)	5 (0.2%)	5 (0.0%)	5 (0.2%)
J96.90	10	Respiratory failure, unspecified, unspecified whether with hypoxia or hypercapnia	5 (0.0%)	4 (0.1%)	4 (0.0%)	3 (0.1%)
D38.1	10	Neoplasm of uncertain behavior of trachea, bronchus and lung	4 (0.0%)	4 (0.1%)	3 (0.0%)	3 (0.1%)

I31.9	10	Disease of pericardium, unspecified	4 (0.0%)	2 (0.1%)	4 (0.0%)	2 (0.1%)
I88.9	10	Nonspecific lymphadenitis, unspecified	4 (0.0%)	4 (0.1%)	3 (0.0%)	3 (0.1%)
D49.1	10	Neoplasm of unspecified behavior of respiratory system	2 (0.0%)	1 (0.0%)	2 (0.0%)	1 (0.0%)
J42	10	Unspecified chronic bronchitis	2 (0.0%)	2 (0.1%)	1 (0.0%)	1 (0.0%)
J81.1	10	Chronic pulmonary edema	2 (0.0%)	2 (0.1%)	2 (0.0%)	2 (0.1%)
518.89	10	Other diseases of lung, not elsewhere classified	1 (0.0%)	1 (0.0%)	1 (0.0%)	1 (0.0%)
C34.30	10	Malignant neoplasm of lower lobe, unspecified bronchus or lung	1 (0.0%)	1 (0.0%)	1 (0.0%)	1 (0.0%)
D14.30	10	Benign neoplasm of unspecified bronchus and lung	1 (0.0%)	1 (0.0%)	1 (0.0%)	1 (0.0%)
Alternative Gastrointestinal Based Diagnoses						
530.81	9	Esophageal reflux	709 (1.6%)	379 (13.6%)	324 (1.2%)	219 (7.9%)
560.9	9	Unspecified intestinal obstruction	48 (0.1%)	25 (0.9%)	20 (0.1%)	12 (0.4%)
K21.9	10	Gastro-esophageal reflux disease without esophagitis	60 (0.1%)	34 (1.2%)	36 (0.1%)	24 (0.9%)
K50.90	10	Crohn's disease, unspecified, without complications	55 (0.1%)	8 (0.3%)	23 (0.1%)	7 (0.3%)
K80.20	10	Calculus of gallbladder without cholecystitis without obstruction	2 (0.0%)	2 (0.1%)	1 (0.0%)	1 (0.0%)
Alternative Infectious Disease Based Diagnoses						

486	9	Pneumonia, organism unspecified	2163 (4.8%)	748 (26.9%)	1537 (5.5%)	591 (21.3%)
466	9	Acute bronchitis	684 (1.5%)	473 (17.0%)	309 (1.1%)	231 (8.3%)
599	9	Urinary tract infection, site not specified	619 (1.4%)	293 (10.5%)	231 (0.8%)	141 (5.1%)
461.9	9	Acute sinusitis, unspecified	524 (1.2%)	383 (13.8%)	173 (0.6%)	151 (5.4%)
465.9	9	Acute upper respiratory infections of unspecified site	381 (0.9%)	303 (10.9%)	171 (0.6%)	144 (5.2%)
473.9	9	Unspecified sinusitis (chronic)	358 (0.8%)	200 (7.2%)	173 (0.6%)	117 (4.2%)
462	9	Acute pharyngitis	352 (0.8%)	276 (9.9%)	139 (0.5%)	125 (4.5%)
490	9	Bronchitis, not specified as acute or chronic	310 (0.7%)	230 (8.3%)	155 (0.6%)	130 (4.7%)
79.99	9	Unspecified viral infection	184 (0.4%)	147 (5.3%)	117 (0.4%)	95 (3.4%)
482.9	9	Bacterial pneumonia, unspecified	148 (0.3%)	63 (2.3%)	91 (0.3%)	47 (1.7%)
513	9	Abscess of lung	148 (0.3%)	86 (3.1%)	122 (0.4%)	75 (2.7%)
491.9	9	Unspecified chronic bronchitis	53 (0.1%)	39 (1.4%)	34 (0.1%)	29 (1.0%)
465.8	9	Acute upper respiratory infections of other multiple sites	25 (0.1%)	20 (0.7%)	12 (0.0%)	10 (0.4%)
790.8	9	Viremia, unspecified	14 (0.0%)	10 (0.4%)	11 (0.0%)	7 (0.3%)
J18.9	10	Pneumonia, unspecified organism	154 (0.3%)	52 (1.9%)	126 (0.5%)	48 (1.7%)
N39.0	10	Urinary tract infection, site not specified	89 (0.2%)	33 (1.2%)	42 (0.2%)	19 (0.7%)
J20.9	10	Acute bronchitis, unspecified	33 (0.1%)	22 (0.8%)	19 (0.1%)	15 (0.5%)
J06.9	10	Acute upper respiratory infection, unspecified	28 (0.1%)	25 (0.9%)	17 (0.1%)	15 (0.5%)

J32.9	10	Chronic sinusitis, unspecified	27 (0.1%)	12 (0.4%)	11 (0.0%)	6 (0.2%)
J01.90	10	Acute sinusitis, unspecified	24 (0.1%)	18 (0.6%)	14 (0.1%)	10 (0.4%)
J02.9	10	Acute pharyngitis, unspecified	17 (0.0%)	13 (0.5%)	11 (0.0%)	9 (0.3%)
J40	10	Bronchitis, not specified as acute or chronic	14 (0.0%)	11 (0.4%)	10 (0.0%)	8 (0.3%)
B34.9	10	Viral infection, unspecified	12 (0.0%)	11 (0.4%)	9 (0.0%)	8 (0.3%)
J15.9	10	Unspecified bacterial pneumonia	4 (0.0%)	3 (0.1%)	3 (0.0%)	2 (0.1%)
B97.89	10	Other viral agents as the cause of diseases classified elsewhere	3 (0.0%)	3 (0.1%)	1 (0.0%)	1 (0.0%)
J85.2	10	Abscess of lung without pneumonia	3 (0.0%)	2 (0.1%)	3 (0.0%)	2 (0.1%)
J85.1	10	Abscess of lung with pneumonia	2 (0.0%)	2 (0.1%)	1 (0.0%)	1 (0.0%)
Symptom Based Diagnoses						
786.5	9	Chest pain, unspecified	1782 (4.0%)	900 (32.4%)	1027 (3.7%)	598 (21.5%)
786.2	9	Cough	1762 (3.9%)	940 (33.8%)	1159 (4.1%)	684 (24.6%)
786.05	9	Shortness of breath	1551 (3.5%)	803 (28.9%)	946 (3.4%)	595 (21.4%)
780.6	9	Fever, unspecified	1147 (2.6%)	496 (17.8%)	917 (3.3%)	416 (15.0%)
780.79	9	Other malaise and fatigue	1085 (2.4%)	614 (22.1%)	562 (2.0%)	386 (13.9%)
789	9	Abdominal pain, unspecified site	1013 (2.3%)	530 (19.1%)	485 (1.7%)	301 (10.8%)
784	9	Headache	693 (1.6%)	342 (12.3%)	322 (1.2%)	202 (7.3%)
780.6	9	Fever and other physiologic disturbances of temperature regulation	499 (1.1%)	196 (7.1%)	362 (1.3%)	155 (5.6%)

786.59	9	Other chest pain	436 (1.0%)	287 (10.3%)	238 (0.9%)	179 (6.4%)
787.91	9	Diarrhea	431 (1.0%)	234 (8.4%)	200 (0.7%)	120 (4.3%)
789.07	9	Abdominal pain, generalized	291 (0.7%)	173 (6.2%)	131 (0.5%)	83 (3.0%)
783.21	9	Loss of weight	271 (0.6%)	162 (5.8%)	185 (0.7%)	119 (4.3%)
787.02	9	Nausea alone	242 (0.5%)	142 (5.1%)	123 (0.4%)	83 (3.0%)
789.01	9	Abdominal pain, right upper quadrant	218 (0.5%)	120 (4.3%)	104 (0.4%)	68 (2.4%)
786.52	9	Painful respiration	209 (0.5%)	152 (5.5%)	143 (0.5%)	106 (3.8%)
789.06	9	Abdominal pain, epigastric	202 (0.5%)	133 (4.8%)	102 (0.4%)	71 (2.6%)
786.3	9	Hemoptysis	107 (0.2%)	45 (1.6%)	76 (0.3%)	30 (1.1%)
786.07	9	Wheezing	84 (0.2%)	69 (2.5%)	40 (0.1%)	35 (1.3%)
786.3	9	Hemoptysis, unspecified	81 (0.2%)	44 (1.6%)	59 (0.2%)	34 (1.2%)
780.8	9	Generalized hyperhidrosis	73 (0.2%)	53 (1.9%)	45 (0.2%)	35 (1.3%)
R06.02	10	Shortness of breath	153 (0.3%)	54 (1.9%)	84 (0.3%)	41 (1.5%)
R05	10	Cough	140 (0.3%)	77 (2.8%)	106 (0.4%)	62 (2.2%)
R50.9	10	Fever, unspecified	112 (0.3%)	41 (1.5%)	101 (0.4%)	37 (1.3%)
R07.9	10	Chest pain, unspecified	98 (0.2%)	53 (1.9%)	64 (0.2%)	39 (1.4%)
R06.00	10	Dyspnea, unspecified	60 (0.1%)	32 (1.2%)	32 (0.1%)	23 (0.8%)
R10.9	10	Unspecified abdominal pain	55 (0.1%)	36 (1.3%)	30 (0.1%)	19 (0.7%)
R07.89	10	Other chest pain	54 (0.1%)	29 (1.0%)	28 (0.1%)	16 (0.6%)
R53.83	10	Other fatigue	40 (0.1%)	28 (1.0%)	27 (0.1%)	21 (0.8%)
R51	10	Headache	27 (0.1%)	17 (0.6%)	19 (0.1%)	13 (0.5%)
R04.2	10	Hemoptysis	26 (0.1%)	9 (0.3%)	17 (0.1%)	8 (0.3%)
R63.4	10	Abnormal weight loss	26 (0.1%)	13 (0.5%)	17 (0.1%)	11 (0.4%)
R19.7	10	Diarrhea, unspecified	20 (0.0%)	12 (0.4%)	11 (0.0%)	6 (0.2%)
R06.09	10	Other forms of dyspnea	18 (0.0%)	11 (0.4%)	9 (0.0%)	7 (0.3%)
R07.81	10	Pleurodynia	18 (0.0%)	12 (0.4%)	13 (0.0%)	8 (0.3%)

R10.11	10	Right upper quadrant pain	18 (0.0%)	10 (0.4%)	6 (0.0%)	5 (0.2%)
R10.13	10	Epigastric pain	15 (0.0%)	12 (0.4%)	8 (0.0%)	7 (0.3%)
R06.2	10	Wheezing	14 (0.0%)	8 (0.3%)	10 (0.0%)	7 (0.3%)
R10.84	10	Generalized abdominal pain	13 (0.0%)	12 (0.4%)	7 (0.0%)	7 (0.3%)
R53.81	10	Other malaise	11 (0.0%)	7 (0.3%)	6 (0.0%)	3 (0.1%)
R06.89	10	Other abnormalities of breathing	10 (0.0%)	6 (0.2%)	5 (0.0%)	4 (0.1%)
R61	10	Generalized hyperhidrosis	10 (0.0%)	9 (0.3%)	7 (0.0%)	6 (0.2%)
R53.1	10	Weakness	9 (0.0%)	7 (0.3%)	6 (0.0%)	4 (0.1%)
R11.0	10	Nausea	7 (0.0%)	6 (0.2%)	3 (0.0%)	3 (0.1%)
R07.1	10	Chest pain on breathing	5 (0.0%)	4 (0.1%)	2 (0.0%)	2 (0.1%)
R06.83	10	Snoring	4 (0.0%)	2 (0.1%)	4 (0.0%)	2 (0.1%)
Testing Imaging or Physical Exam Based Diagnoses						
786.6	9	Swelling, mass, or lump in chest	2263 (5.1%)	862 (31.0%)	1817 (6.5%)	767 (27.6%)
785.6	9	Enlargement of lymph nodes	1915 (4.3%)	778 (28.0%)	1531 (5.5%)	678 (24.4%)
793.1	9	Lung field	999 (2.2%)	554 (19.9%)	731 (2.6%)	446 (16.0%)
793.19	9	Other nonspecific abnormal finding of lung field	956 (2.1%)	440 (15.8%)	726 (2.6%)	381 (13.7%)
285.9	9	Anemia, unspecified	887 (2.0%)	366 (13.2%)	429 (1.5%)	226 (8.1%)
793.11	9	Solitary pulmonary nodule	777 (1.7%)	313 (11.3%)	621 (2.2%)	282 (10.1%)
555.9	9	Regional enteritis of unspecified site	698 (1.6%)	86 (3.1%)	277 (1.0%)	71 (2.6%)
511.9	9	Unspecified pleural effusion	449 (1.0%)	244 (8.8%)	328 (1.2%)	194 (7.0%)
518	9	Pulmonary collapse	405 (0.9%)	320 (11.5%)	310 (1.1%)	250 (9.0%)
518.3	9	Pulmonary eosinophilia	359 (0.8%)	219 (7.9%)	272 (1.0%)	179 (6.4%)

783.21	9	Loss of weight	271 (0.6%)	162 (5.8%)	185 (0.7%)	119 (4.3%)
287.5	9	Thrombocytopenia, unspecified	251 (0.6%)	110 (4.0%)	150 (0.5%)	81 (2.9%)
276.1	9	Hyposmolality and/or hyponatremia	223 (0.5%)	117 (4.2%)	144 (0.5%)	90 (3.2%)
799.02	9	Hypoxemia	213 (0.5%)	89 (3.2%)	108 (0.4%)	67 (2.4%)
289.3	9	Lymphadenitis, unspecified, except mesenteric	180 (0.4%)	137 (4.9%)	139 (0.5%)	114 (4.1%)
784.2	9	Swelling, mass, or lump in head and neck	169 (0.4%)	90 (3.2%)	89 (0.3%)	53 (1.9%)
729.81	9	Swelling of limb	167 (0.4%)	134 (4.8%)	82 (0.3%)	67 (2.4%)
429.3	9	Cardiomegaly	145 (0.3%)	120 (4.3%)	79 (0.3%)	70 (2.5%)
512.8	9	Other pneumothorax and air leak	143 (0.3%)	87 (3.1%)	108 (0.4%)	74 (2.7%)
574.2	9	Calculus of gallbladder without mention of cholecystitis, without mention of obstruction	142 (0.3%)	93 (3.3%)	74 (0.3%)	51 (1.8%)
789.2	9	Splenomegaly	141 (0.3%)	95 (3.4%)	106 (0.4%)	76 (2.7%)
V74.1	9	Screening examination for pulmonary tuberculosis	138 (0.3%)	124 (4.5%)	73 (0.3%)	70 (2.5%)
288.6	9	Leukocytosis, unspecified	113 (0.3%)	72 (2.6%)	62 (0.2%)	45 (1.6%)
284.19	9	Other pancytopenia	97 (0.2%)	39 (1.4%)	65 (0.2%)	29 (1.0%)
790.4	9	Nonspecific elevation of levels of transaminase or lactic acid dehydrogenase [LDH]	95 (0.2%)	62 (2.2%)	62 (0.2%)	39 (1.4%)
512.89	9	Other pneumothorax	89 (0.2%)	57 (2.1%)	76 (0.3%)	49 (1.8%)

782.2	9	Localized superficial swelling, mass, or lump	75 (0.2%)	62 (2.2%)	45 (0.2%)	37 (1.3%)
R91.8	10	Other nonspecific abnormal finding of lung field	315 (0.7%)	99 (3.6%)	235 (0.8%)	92 (3.3%)
R91.1	10	Solitary pulmonary nodule	212 (0.5%)	69 (2.5%)	162 (0.6%)	63 (2.3%)
R59.0	10	Localized enlarged lymph nodes	77 (0.2%)	41 (1.5%)	62 (0.2%)	34 (1.2%)
J90	10	Pleural effusion, not elsewhere classified	57 (0.1%)	22 (0.8%)	48 (0.2%)	19 (0.7%)
R22.2	10	Localized swelling, mass and lump, trunk	38 (0.1%)	20 (0.7%)	27 (0.1%)	17 (0.6%)
R59.1	10	Generalized enlarged lymph nodes	35 (0.1%)	19 (0.7%)	29 (0.1%)	19 (0.7%)
R59.9	10	Enlarged lymph nodes, unspecified	29 (0.1%)	22 (0.8%)	24 (0.1%)	19 (0.7%)
J98.11	10	Atelectasis	26 (0.1%)	23 (0.8%)	18 (0.1%)	17 (0.6%)
R63.4	10	Abnormal weight loss	26 (0.1%)	13 (0.5%)	17 (0.1%)	11 (0.4%)
R09.02	10	Hypoxemia	18 (0.0%)	8 (0.3%)	10 (0.0%)	6 (0.2%)
J93.9	10	Pneumothorax, unspecified	16 (0.0%)	13 (0.5%)	13 (0.0%)	12 (0.4%)
D72.829	10	Elevated white blood cell count, unspecified	15 (0.0%)	7 (0.3%)	10 (0.0%)	6 (0.2%)
E871	10	Foreign object left in body during procedure	13 (0.0%)	10 (0.4%)	11 (0.0%)	9 (0.3%)
R16.1	10	Splenomegaly, not elsewhere classified	13 (0.0%)	9 (0.3%)	13 (0.0%)	9 (0.3%)
D61.818	10	Other pancytopenia	12 (0.0%)	3 (0.1%)	6 (0.0%)	3 (0.1%)

D69.6	10	Thrombocytopenia, unspecified	12 (0.0%)	7 (0.3%)	9 (0.0%)	6 (0.2%)
Z11.1	10	Encounter for screening for respiratory tuberculosis	11 (0.0%)	9 (0.3%)	3 (0.0%)	3 (0.1%)
I51.7	10	Cardiomegaly	9 (0.0%)	6 (0.2%)	7 (0.0%)	5 (0.2%)
R22.1	10	Localized swelling, mass and lump, neck	7 (0.0%)	3 (0.1%)	6 (0.0%)	2 (0.1%)
R22.0	10	Localized swelling, mass and lump, head	3 (0.0%)	3 (0.1%)	3 (0.0%)	3 (0.1%)
R74.0	10	Nonspecific elevation of levels of transaminase and lactic acid dehydrogenase [LDH]	2 (0.0%)	2 (0.1%)	2 (0.0%)	2 (0.1%)

Supplementary Table S3 - List of Respiratory Antibiotics

Levofloxacin, Ciprofloxacin, Azithromycin, Cephalexin, Amoxicillin, Doxycycline, Moxifloxacin, Clindamycin, Vancomycin, Cefuroxime, Clarithromycin, Cefdinir, Penicillin, Ampicillin, Tetracycline, Erythromycin, Minocycline, Linezolid, Cefpodoxime, Cefaclor, Cefprozil, Dicloxacillin, Cefditoren, Cefixime, Ceftibuten, Telithromycin, Cefazolin, Cefonicid, Cefotetan, Cefoxitin, Loracarbef, Cefotaxime, Ceftizoxime, Ceftriaxone, Cefoperazone, Ceftazidime, Cefepime, Ceftaroline, Ceftolozane, Aztreonam, Imipenem, Meropenem, Ertapenem, Doripenem, Tazobactam, Sulfamethoxazole

Supplementary Table S4 – ICD and CPT codes used in risk factor analysis

Category	Codes (ICD/CPT/CCS)
Asthma	*<u>ICD-9-CM</u>:* 493.00, 493.01, 493.02, 493.10, 493.11, 493.12, 493.20, 493.21, 493.22, 493.81, 493.82, 493.90,493.92<br><br>*<u>ICD-10-CM</u>:* J45.20, J45.21, J45.22, J45.30, J45.31, J45.32, J45.40, J45.41, J45.42, J45.50, J45.51, J45.52, J45.901, J45.902, J45.909, J45.990, J45.991, J45.998
COPD	*<u>ICD-9-CM</u>:* 490, 491.0, 491.1, 491.8, 491.9<br><br>*<u>ICD-10-CM</u>:* J40, J41.0, J41.1, J42, J44.0, J47.0
IPD	*<u>ICD-9-CM</u>:* 516.3, 516.30, 516.31, 516.32, 516.33, 516.34, 516.35, 516.36, 516.37<br><br>*<u>ICD-10-CM</u>:* J84, J84.0, J84.01, J84.02, J84.03, J84.09, J84.1, J84.10, J84.11, J84.111, J84.112, J84.113, J84.114, J84.115, J84.116, J84.117, J84.17, J84.2, J84.8, J84.81, J84.82, J84.83, J84.84, J84.841, J84.842, J84.843, J84.848, J84.89, J84.9
HIV (CCS code)	5
Chest – CT (CPT codes)	71260, 71250, 71270
Chest – Xray (CPT codes)	71010, 71015, 71020, 71021, 71022, 71023, 71030, 71034, 71035, 71101, 71111, 71120,71045, 71046, 71047, 71048

Supplementary Table S5 – Regression results for likelihood of experiencing a delay based on various geographic models

Variable	Effect Estimate	95% CI	P-value
Using Metropolitan Statistical Area (MSA) Case Counts in Truven			
Top 5% of MSAs	0.947	0.852, 1.052	0.312
Top 10% of MSAs	0.897	0.807, 0.996	0.043
Top 15% of MSAs	0.933	0.835, 1.042	0.220
Top 20% of MSAs	0.950	0.845, 1.069	0.395
Top 1 MSAs	0.910	0.717, 1.153	0.434
Top 2 MSAs	0.850	0.715, 1.010	0.065
Top 3 MSAs	0.879	0.755, 1.023	0.096
Top 4 MSAs	0.882	0.768, 1.013	0.075
Top 5 MSAs	0.931	0.818, 1.059	0.278
Top 10 MSAs	0.955	0.855, 1.068	0.421
Top 20 MSAs	0.927	0.835, 1.029	0.155
Top 30 MSAs	0.899	0.810, 0.998	0.046
Top 50 MSAs	0.933	0.835, 1.042	0.220
Top 100 MSAs	0.990	0.868, 1.129	0.877
Using Weighted Annual MSA Incidence in Marketscan Data			
Top 5% of MSAs	0.878	0.504, 1.528	0.645
Top 10% of MSAs	1.035	0.792, 1.354	0.799
Top 15% of MSAs	1.016	0.829, 1.245	0.877
Top 20% of MSAs	1.023	0.853, 1.225	0.809
Top 1 MSAs	0.878	0.190, 4.050	0.868
Top 2 MSAs	0.862	0.220, 3.380	0.831
Top 3 MSAs	0.914	0.275, 3.042	0.884
Top 4 MSAs	1.012	0.343, 2.987	0.983
Top 5 MSAs	1.114	0.413, 3.001	0.831
Top 10 MSAs	1.137	0.566, 2.285	0.718
Top 20 MSAs	1.025	0.627, 1.676	0.921
Top 30 MSAs	0.960	0.711, 1.296	0.789
Top 50 MSAs	1.013	0.828, 1.240	0.901
Top 100 MSAs	0.990	0.871, 1.126	0.882
Using Incidence Based on Map from Baddley JW, et al. (Geographic distribution of endemic fungal infections among older persons, United States. *Emerg Infect Dis.* 2011 Sep;17(9):1664-9.)			
0.55 - 0.99	REF	REF	REF
1.14 -1.86	1.145	0.906, 1.449	0.258
2.07 - 3.06	1.048	0.855, 1.283	0.653

3.26 - 5.19	1.087	0.907, 1.303	0.366
5.60 - 7.39	0.995	0.824, 1.202	0.959
7.84 - 12.30	1.019	0.855, 1.216	0.830
Using Regions Based on US Census Bureau Regions and Divisions by State			
Midwest	REF	REF	REF
Northeast	1.088	0.874, 1.356	0.450
South	0.973	0.886, 1.069	0.573
West	0.925	0.723, 1.183	0.533
Using Weighted Annual State Incidence in Marketscan			
Incidence > 40/100,000	0.962	0.880, 1.051	0.389
Using CDC Reportable States			
CDC Reportable States (AR, DE, IL, IN, KS, KY, LA, MI, MN, NE, PA, RI, and WI)	0.976	0.893, 1.067	0.598

Note: Models were adjusted for all variables in Table 3. MSA = metropolitan statistical area.

Supplementary Table S6 - Count of symptomatically similar diagnoses (SSDs) by category

SSD	Entire Study Period (1-365 Days Prior to histoplasmosis diagnosis)		Delay Opportunity Window (1-97 Days Prior to histoplasmosis diagnosis)	
	Total Patients with SSD (% of all patients)	Total Visits with SSD (% of all visits in window)	Total Patients with SSD (% of all patients)	Total Visits with SSD (% of all visits in window)
Alternative-Gastrointestinal-Based Diagnoses	437 (15.4%)	929 (1.1%)	260 (9.1%)	431 (0.5%)
Alternative-Infectious-Disease-Based Diagnoses	1911 (67.2%)	6588 (7.6%)	1349 (47.5%)	3681 (4.0%)
Alternative-Cardiopulmonary-Based Diagnoses	1916 (67.4%)	10103 (11.6%)	1734 (61.0%)	6184 (6.8%)
Testing-, Imaging-, or Physical-Exam-Based	2188 (77.0%)	11652 (13.4%)	1980 (69.7%)	7991 (8.7%)
Symptom-Based Diagnoses	2385 (83.9%)	12105 (13.9%)	1990 (70.0%)	7119 (7.8%)
All SSDS	2727 (96.0%)	32140 (36.9%)	2562 (90.1%)	18886 (20.7%)

Note: SSD = Symptomatically Similar Diagnosis.

Supplementary Table S7 – Regression model stratified by ICD-9/10 index diagnosis

Variable	**ICD-9-CM Index Diagnosis (n Patients = 2,592)** (OR(CI); p-value)	**ICD-10-CM Index Diagnosis (n Patients = 250)** (OR(CI); p-value)
**Weekend (visits that occurred on a Saturday or Sunday)**	1.829 (1.482, 2.257); <0.001	2.118 (0.931, 4.821); 0.074
**Female Sex**	0.995 (0.908, 1.09); 0.912	0.787 (0.528, 1.173); 0.239
**Age**		
< 18	REF	REF
18 - 35	1.106 (0.874, 1.398); 0.401	0.421 (0.140, 1.268); 0.124
36 - 45	1.171 (0.930, 1.475); 0.180	0.382 (0.123, 1.190); 0.097
46 - 55	1.206 (0.966, 1.505); 0.098	0.501 (0.161, 1.561); 0.234
56 - 65	1.193 (0.956, 1.489); 0.118	0.783 (0.261, 2.346); 0.661
>65	1.281 (1.005, 1.633); 0.046	0.772 (0.243, 2.454); 0.661
**Settings visited**		
Outpatient only	REF	REF
All three (inpatient, outpatient, and ED)	0.164 (0.104, 0.257); <0.001	0.054 (0.003, 0.860); 0.039
ED only	11.28 (5.329, 23.878); <0.001	1.619 (0.615, 4.262); 0.329
Inpatient only	0.131 (0.111, 0.153); <0.001	0.084 (0.044, 0.164); <0.001
Inpatient and ED	0.146 (0.107, 0.199); <0.001	0.291 (0.056, 1.520); 0.143
Inpatient and outpatient	0.132 (0.112, 0.156); <0.001	0.139 (0.071, 0.271); <0.001
Outpatient and ED	2.596 (1.625, 4.147); <0.001	8.564 (1.117, 65.631); 0.039
**Urban vs. not urban**	1.040 (0.936, 1.156); 0.469	0.646 (0.412, 1.012); 0.057
**Asthma prior to change point**	1.160 (0.977, 1.376); 0.090	1.072 (0.490, 2.348); 0.861
**COPD prior to change point**	1.316 (1.140, 1.518); <0.001	1.119 (0.622, 2.015); 0.707
**IPD prior to change point**	1.627 (0.829, 3.194); 0.157	NA
**HIV prior to index**	0.845 (0.669, 1.068); 0.159	1.014 (0.113, 9.070); 0.990
**Chest CT prior to change point**	1.597 (1.447, 1.762); <0.001	2.165 (1.437, 3.262); <0.001
**Chest X-Ray prior to change point**	2.283 (2.045, 2.548); <0.001	3.706 (2.428, 5.655); <0.001
**Respiratory antibiotics between change point and 1 days prior to index**	1.278 (1.162, 1.404); <0.001	1.441 (0.970, 2.141); 0.07

Note: Model was also adjusted for year and month of SSD/index visit. ED = emergency department.

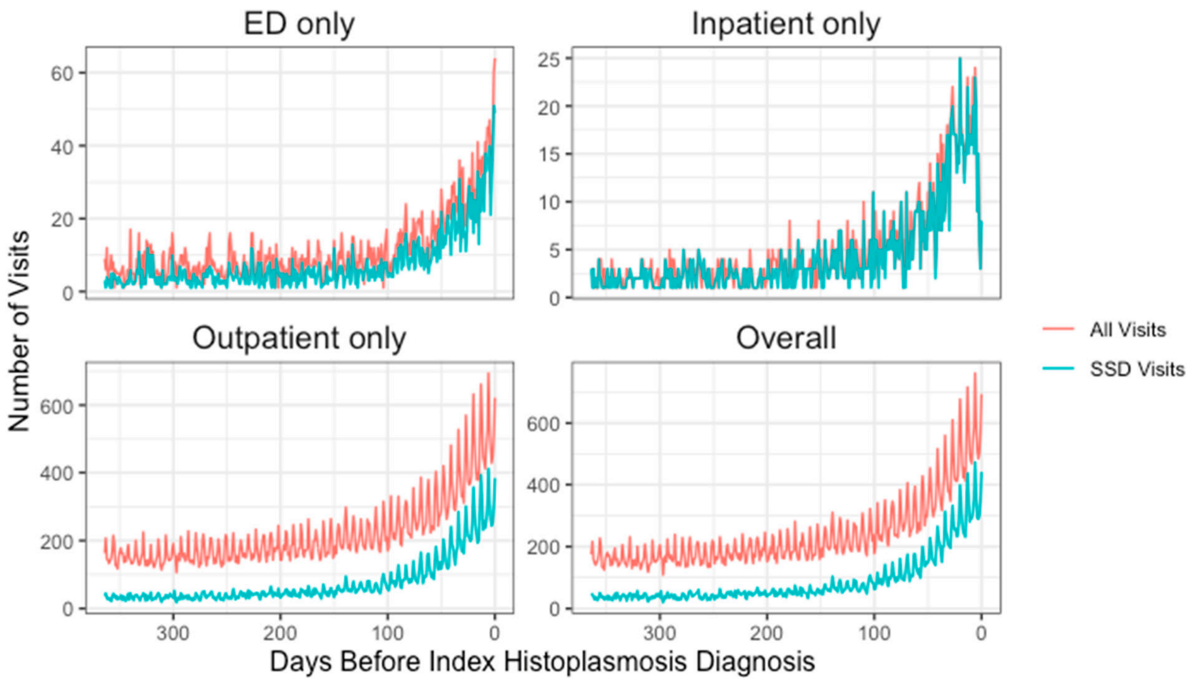

Supplementary Figure S1 – Trend in symptomatically similar diagnosis (SSD) visits and all visits prior to the index histoplasmosis diagnoses broken down by type of healthcare setting. The red lines depict all visits, and the blue line depicts visits with SSD-related conditions

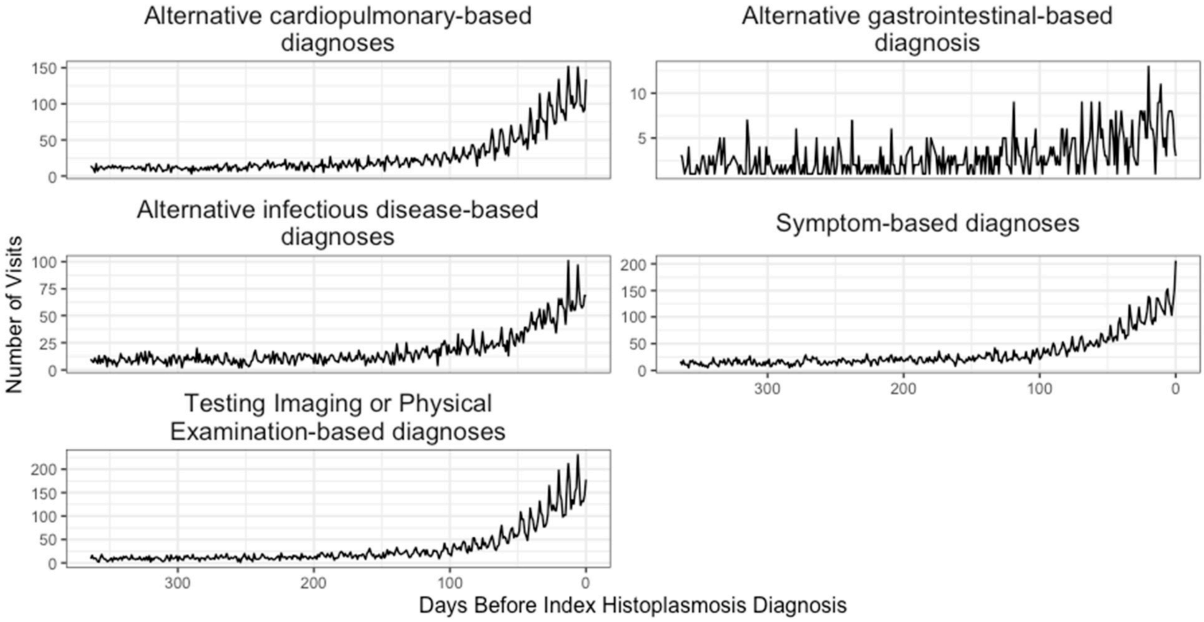

Supplementary Figure S2 – Trend in symptomatically similar diagnosis (SSD) visits prior to diagnosis for the five SSD categories
